# Feasibility assessment of crowdsourcing slogans for promoting household waste segregation in India: a cross-sectional study

**DOI:** 10.3389/fpubh.2023.1118331

**Published:** 2023-10-11

**Authors:** Kavya Krishnan, Krushna Chandra Sahoo, Madhanraj Kalyanasundaram, Surya Singh, Asha Srinivas, Ashish Pathak, Cecilia Stålsby Lundborg, Salla Atkins, Kamran Rousta, Vishal Diwan

**Affiliations:** ^1^Division of Environmental Monitoring and Exposure Assessment (Water and Soil), ICMR—National Institute for Research in Environmental Health, Bhopal, India; ^2^ICMR—Regional Medical Research Centre, Bhubaneswar, India; ^3^ICMR—National Institute of Epidemiology, Chennai, India; ^4^Gram Seva Sangh, Bengaluru, India; ^5^Department of Pediatrics, R D Gardi Medical College, Ujjain, India; ^6^Department of Global Public Health, Health Systems and Policy (HSP): Improving Use of Medicines, Karolinska Institutet, Stockholm, Sweden; ^7^Department of Global Public Health, Social Medicine Infectious Disease and Migration (SIM), Karolinska Institutet, Stockholm, Sweden; ^8^Global Health and Development, Faculty of Social Sciences, Tampere University, Tampere, Finland; ^9^Swedish Centre for Resource Recovery, University of Borås, Borås, Sweden

**Keywords:** crowd sourcing, online contest, household waste segregation, India, slogan contest, environmental health, community participation

## Abstract

**Introduction:**

Crowdsourcing is an emerging technique to engage or access a wider set of experts and multiple stakeholders through online platforms, which might effectively be employed in waste management. Therefore, we assessed the feasibility of the crowdsourcing method to provide an alternative approach that can improve household waste segregation using an “online-slogan-contest”.

**Methods:**

The contest was promoted via targeted emails to various governmental and non-governmental organizations and through social media platforms for around 4 weeks (25 days). The entries were received through a Google form. The slogans were assessed by the experts and analyzed using content analysis methods.

**Results:**

Total 969 entries were received from different geographic regions in India. Of that, 456 were in English and 513 in Hindi. Five themes of waste segregation emerged from the received slogans: (1) Community awareness, responsibility, and support, (2) Significance of household waste segregation, (3) Use of separate dustbins, (4) Health and well-being, and (5) Environment and sustainability.

**Discussion:**

Crowdsourcing approaches can be used by local authorities for improving waste management approaches and are recommended as these involve a wider audience within a short time frame. Moreover, this approach is flexible and integrating crowdsourcing approaches strengthens our understanding of existing waste management activities.

## Introduction

Despite the considerable health and environmental consequences of inadequate waste disposal (1), community engagement in segregating and recycling solid waste remains limited due to lack of awareness regarding the long-term benefits of recycling. Robust implementations are required to sensitize the public, and enhance public awareness and participation. Social media and other internet channels exert significant influence on society, making public sensitization easier. Crowdsourcing is an emerging strategy (2) that engages a large group of individuals to collect their insights and concepts on various subjects or to address a societal problem by utilizing online resources (3).

Crowdsourcing is “when a firm outsources some functions to an unspecified group through an open call”. It involves people with varied skills and ideas joining willingly for monetary remuneration or skill improvement (2, 4, 5). It involves people with varied skills and ideas joining willingly for monetary remuneration or skill improvement (5). It saves money, time, and labor (5, 6). It is used for public health, disaster management, environmental issues, waste management, crime reporting, public safety, road safety, and smart city infrastructure. People’s ability to think, communicate, and solve problems is improved by involving them in data collection (7, 8). Crowdsourcing in health communication is supposed to help engage more people (4). Some researchers have also identified health social innovations through crowdsourcing (9). Disease diagnosis, surveillance, environmental health assessment, health education, psychology, etc., have used crowdsourcing (8). Online open challenge contests gather the public’s views on specific topics. Crowdsourcing contests are commonly used in public health research to increase public awareness and community engagement (10). Crowdsourcing-based contests were effectively employed in various public health studies (11, 12). Crowdsourcing was used to develop interventions for alcohol use disorder in the United States (13), psychosocial interventions for providing support to Alzheimer’s Disease Caregivers (14), hurricane-affected areas in Florida (15), and so on. All this research commented on the acceptability of crowdsourcing approaches and their future applicability in resolving public health problems by creating innovative remedies.

Waste management is another important field where crowdsourcing can be effectively employed (7). Crowdsourcing is successfully implemented as a tool for mapping and getting information regarding waste disposals (7). Mobile crowdsourcing (MCS) in smart cities promotes urban planning activities; the public becomes an active player as a data generator in smart city development (16). Many Indian cities have initiated mobile-based applications and WhatsApp groups to involve the public in the municipal system and other initiatives. Crowdsourcing is used to map and gather waste disposal information (7, 16).

It is a potential technique for generating feasible ideas for recurring solid waste management problems (17). Studies have demonstrated the efficacy of online media in conveying education and awareness to the intended audience, hence fostering motivation and behavior modification in the intended audience (17, 18). Crowdsourcing engages a larger audience to disseminate information and messages regarding numerous topical issues efficiently. It can be used effectively in situations with limited resources because it is a simple and time-saving method for reaching a broader audience.

Crowdsourcing has grown in popularity in India as a means of harnessing the power of the masses (19) and leveraging collective intelligence to solve complex problems (20). These platforms raise awareness, promote transparency, and encourage citizen involvement in the fight against social issues (21, 22). It has been useful in addressing social issues; for example, platforms such as “Swechha” enable citizens to report environmental issues (23). Furthermore, the Indian government has launched initiatives such as “MyGov” and “Smart City Challenge,” in which citizens can contribute ideas, suggestions, and feedback on various public policies and urban development projects (24, 25). It has also proven useful in times of crisis and natural disasters (26).

Crowd sourcing is a problem-solving and production paradigm that uses the collective intellect of networked groups to achieve specific goals. Crowdsourcing based approaches can be effectively integrated into various public health researches but the utility of crowdsourcing tool to address public health issues are still in its infancy in India. The current study was conducted as a part of a Swedish Research Council for Environment, Agricultural Sciences and Spatial Planning (FORMAS) funded project focusing on a community-based cluster randomized controlled trial to assess the effectiveness of improved information and volunteer support on segregation of solid waste at the household level in Ujjain City, Madhya Pradesh (I-MISS) (27). In this study we assessed the feasibility of the crowdsourcing method in terms of reach (place/person distribution), richness in the idea (explored using qualitative approach) on household waste segregation via an “online slogan contest.” It was also envisaged to utilize the received slogans and other study findings in the development of waste segregation communication tool for IMISS Project.

## Methods

**Figure 1:**
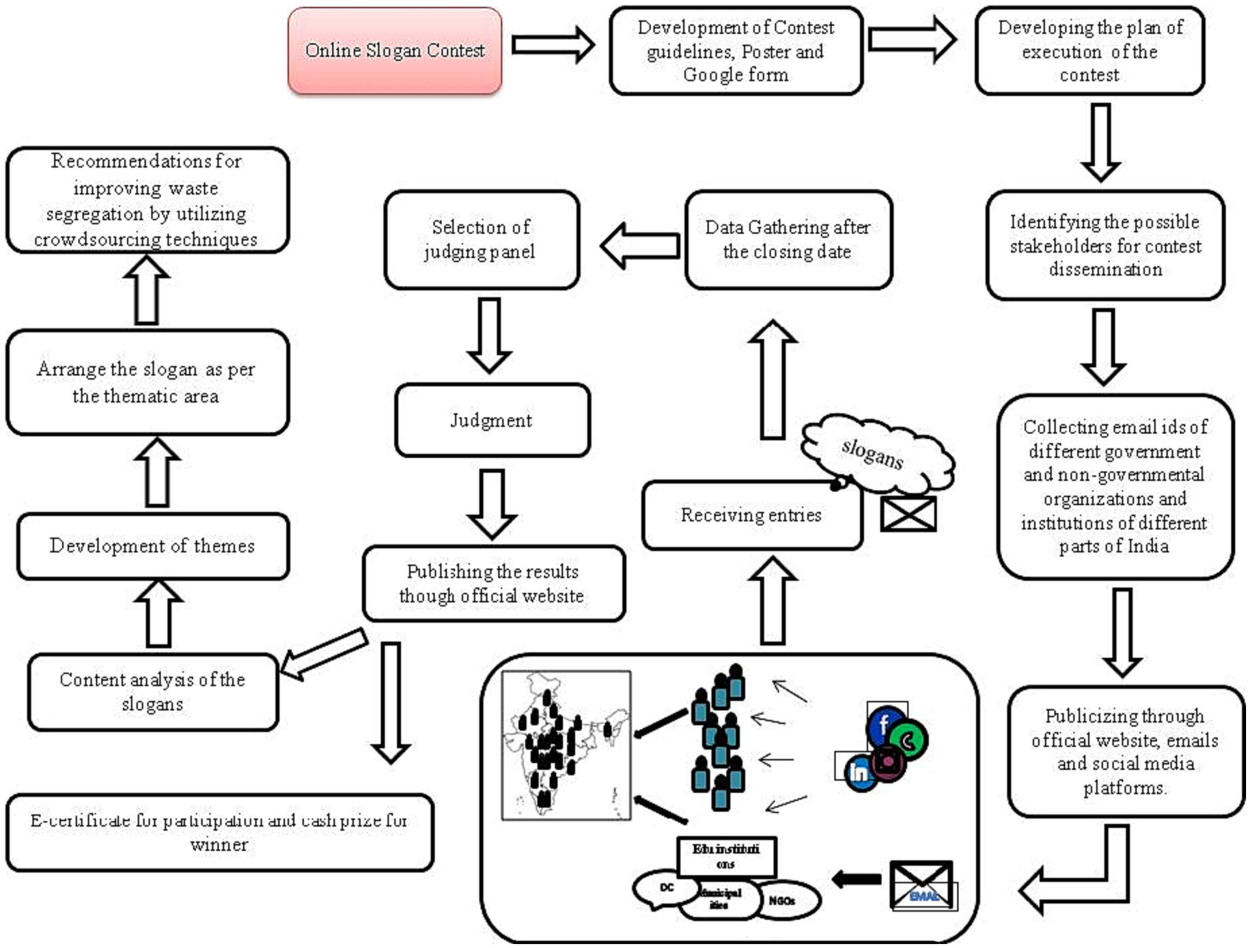
Schematic flow diagram for the detailed crowdsourcing approaches.

We used crowdsourcing-based online slogan contest to explore the key message for awareness on better household waste segregation in India. The slogan contest was hosted by ICMR-National Institute for Research in Environmental Health (NIREH), Bhopal, in collaboration with R D Gardi Medical College, Ujjain, and Ujjain Municipal Corporation. Figure 1 depicts the schematic flow diagram for the detailed crowdsourcing approaches followed.

### Promotion, dissemination, and participation

A contest poster and guidelines were developed to disseminate the contest information (Supplementary material 1). The contest was publicized through the official website of the IMISS project^1^ and ICMR-NIREH.^2^ To promote the contest, we collected email addresses of various governmental and non-governmental organizations across India, including educational institutions, municipalities, district collectors, NGOs and departments under Swachh Bharat Mission (SBM). The contest was promoted via targeted emails to respective organizations and social media platforms (WhatsApp, Facebook, Instagram, LinkedIn, etc.). The contest was initiated on March 7th and lasted for around 4 weeks (25 days), till March 31st, 2022. The first round of contest promotion via email was started 1 week before the contest start date, and a final round of promotion was done during the last week of the contest. Social media promotion was continued throughout the contest timeframe. There were no age, or gender restrictions for the submission, and it was open to all Indian citizens. Participants were restricted to submit only one slogan in English or Hindi with a word limit of 15 words.

### Procedure and data organization

The information regarding the “online slogan contest” was posted on the official website of the IMISS project. All information was provided in English and Hindi to reach out to maximum people. The submissions were received through a google form with socio-demographic details (participant name, age, location, education, etc.) and basic information on participants’ knowledge regarding household waste segregation and disposal practices such as their participation in waste segregation, responsibility of waste segregation and methods of disposal. The google form link was attached to the contest poster, which was disseminated through email and social media. All the entries were automatically recorded with the respective date and time of submission. Participants could win based on the expert assessment, and monetary incentives were decided to be given to the top three participants, separately for English and Hindi slogans.

After the submission deadline, all the entries were collected from the google form. We gave a unique code to each participant and their identities were not revealed to the judges to avoid judgment bias. The entries were categorized into two; participants who submitted Hindi slogans and participants who submitted English slogans. The entries that were irrelevant to the contest and those that exceeded the word limit (15 words) were excluded from the list. The entries with more than one slogan and duplicate slogans were also excluded. The final list was sent for the assessment.

### Judgment of the information

A list of 10 probable stakeholders with experience in waste management and research activities was prepared, of that three experts (two female and one male) were selected. The judging panel included a medical doctor having experience in teaching and public health research, a principal scientist and advisor of a prominent NGO focusing on waste management, and a public health scientist with experience in institutional waste management activities. The judges assessed the slogans according to a set criteria, i.e., creativity, originality, and relevance to the theme. Each criterion held a maximum of 5 marks. English and Hindi slogans were separately assessed. Regarding an event of a score tie, a second round of assessment was done with a different judging panel. After the judgment, the three participants who scored maximum were finalized as the winners of the slogan contest. Based on the assessment score, the top three slogans were selected. Six participants (three each from English and Hindi entries) were finalized as winners. The result was published on the official website of the IMISS project and ICMR-NIREH. The monetary incentives for the first three positions were INR 5,000, 3,000 and 2,000, respectively. The winners were contacted through email and were awarded cash prizes and achievement certificates. Moreover, all the participants of the contest were provided with participation certificates via email.

### Data management and analysis

Descriptive statistics was used to analyze the socio-demographic information of the participants of the contest. The content analysis approach was used to identify and organize the slogans into various thematic areas. Each slogan was carefully read and assigned to one category based on its content. This process involved multiple coders to ensure intercoder reliability. If disagreements occurred, coders would discuss and resolve it through consensus. First, we identified the condensed meaning unit for each slogan and coded them—two authors (KCS and KK) open coded the slogan and it was cross-checked by VD and MK. As the coding process continued, researchers refined/revised the coding scheme if new themes or categories emerged from the slogans that were not initially considered. This iterative process ensured that the analysis remained flexible and captures the richness of the data. All similar codes were grouped into five major thematic areas based on their similar characteristics. The themes derived solely from the submitted content-based slogan. Then all the slogans were organized as per the themes. After coding all the slogans, the researchers analyzed the data to examine the distribution of slogans across different thematic areas.

### Ethical considerations

The study was approved by Institutional Ethical Committee of National Institute for Research in Environmental Health, Bhopal (NIREH/BPL/IEC/2020–21/41 dated April 21, 2020) and Institutional ethics committee of R D Gardi Medical College, Ujjain (03/2020 dated March 12, 2020).

## Results

**Figure 2:**
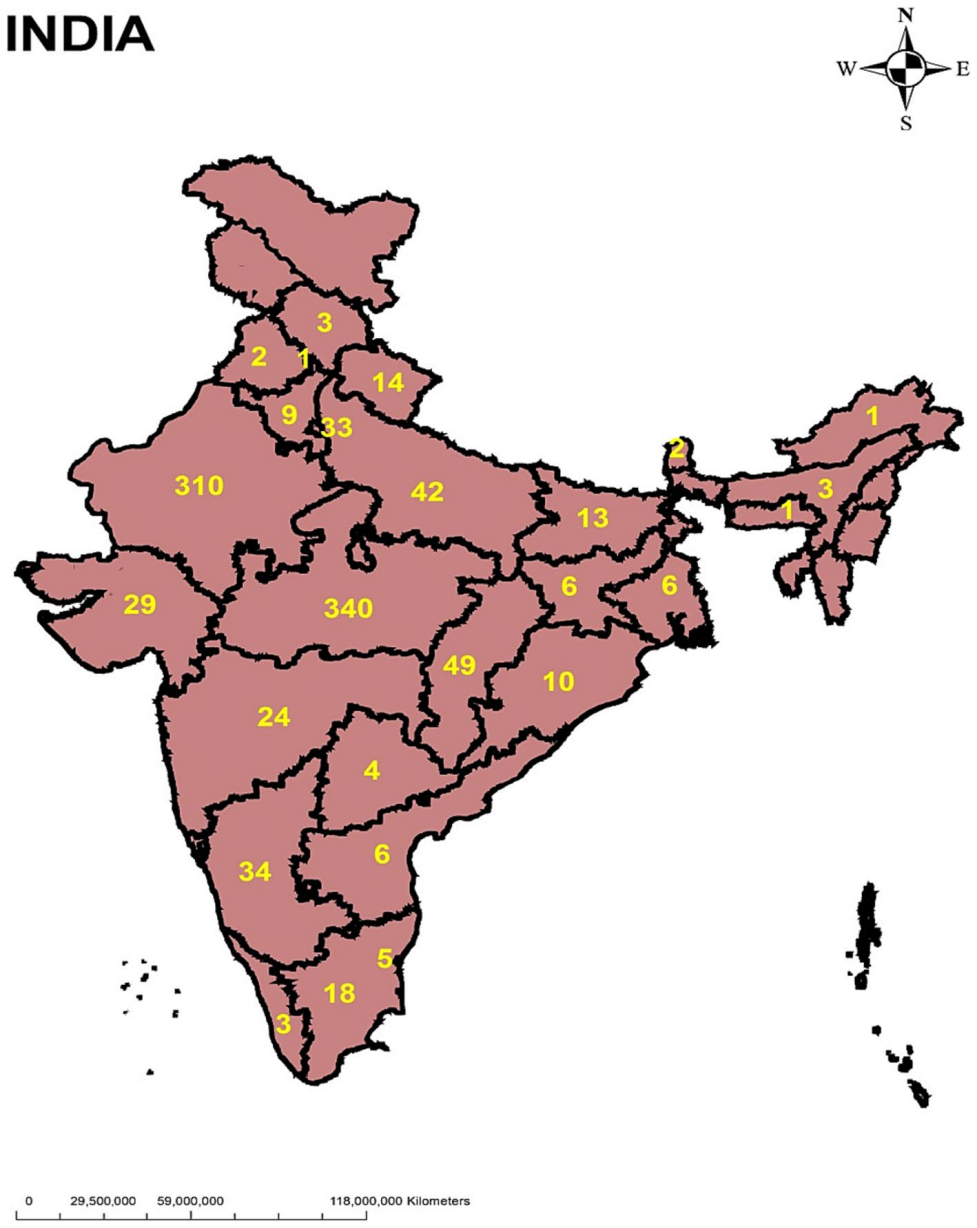
Map showing the participant distribution by state across the country.

A total of 969 entries were received from 23 states and 3 union territories (from total 28 states and 8 union territories) of India (Figure 2). Of that, 456 were in English and 513 in Hindi. Most entries were received from the state of Madhya Pradesh (35.5%) followed by Rajasthan (31.9%). There were 53% male participants and 47% female participants and most participants were from urban areas (70%). More number of participations were students (64.8%), and 30.3% of total participants had an education level of post-graduation and above. The characteristics of participant demographics are given in Table 1.

**Table 1:** Socio-demographic information of the contest participants (*N* = 969).

Variable		No. of entries *n* (%)
Referral source	Social media (WhatsApp and other social media platforms)	369 (38)
	Email	369 (38)
	Website	231 (24)
Location	Madhya Pradesh	340 (35.1)
	Rajasthan	310 (32)
	Chhattisgarh	49 (5.1)
	Uttar Pradesh	42 (4.33)
	Uttarakhand	14 (1.4)
	Karnataka	34 (3.51)
	Delhi	33 (3.41)
	Gujarat	29 (3)
	Maharashtra	24 (2.45)
	Others	94 (9.7)
Age	10–15 years	243 (26)
	16–25 years	270 (29)
	26–39 years	250 (27)
	40 and above	170 (18)
	Mean age (SD) in years	26.6 (13.37)
Gender	Male	511 (53)
	Female	454 (47)
Education	Post-graduate and above	294 (30.3)
	Graduation	248 (25.6)
	Higher secondary	178 (18.4)
	High school	156 (16.1)
	Basic level of education/primary education	92(9.5)
	Illiterate	1 (0.1)
Occupation	Not employed	70 (7)
	Students	558 (58)
	Government service	224 (23)
	Private service	72 (7)
	Self-employed	38 (4)
	Retired	7 (1)
Residence	Urban	687 (71)
	Rural	282 (29)

The received slogans were divided into five thematic areas of waste segregation: (1) Community awareness, responsibility, and support, (2) Significance of household waste segregation, (3) Use of separate dustbin, (4) Health and wellbeing, and (5) Environment and sustainability. The details of first three winning slogans are given in Table 2. A total of 288 relevant slogans were obtained: 82 were related to theme 1 (37 in English and 45 in the Hindi language); 65 to theme 2 (33 in English and 32 in the Hindi language); 69 to theme 3 (13 in English and 56 in the Hindi language); 24 to theme 4 (8 in English and 16 in the Hindi language) and 48 to theme 5 (27 in English and 21 in the Hindi language). The number of slogans in each domain is provided in Table 3. The detailed lists of slogans in relation to household waste segregation in English and Hindi language are presented in Supplementary material 2.

**Table 2:** Winning slogans for English and Hindi language and its thematic areas.

Sl. no	Winning slogans	Thematic area
English		
1	Solid, Dry & Sanitary, Segregation of Waste is Mandatory	Significance of waste segregation
2	Segregate, separate and refuse the refuse also remember, two trash cans to use!	Use of separate dustbin
3	Sustain your own nation - by practicing- household waste segregation	Community awareness, responsibility and support, Significance of waste segregation
Hindi		
1	घर की सफाई में ना करे ढिलाई, कूड़ा अलग करने में है सबकी भलाई।	Health and well-being, Use of separate dustbin
2	कचरे का है एक ही काट, गीले-सूखे में उसे दो तुम बांट।	Use of separate dustbin
3	घरेलू कचरे की करो छटाई, तभी होगी पूर्ण सफाई।	Use of separate dustbin

**Table 3:** Number of slogans in relation to household waste segregation in English and Hindi language.

Slogans	Total *n* (%)	Thematic areas				
		Community awareness, responsibility and support *n* (%)	Significance of household waste segregation *n* (%)	Use of separate dustbin *n* (%)	Health and wellbeing *n* (%)	Environment and sustainability *n* (%)
English	118 (41)	37 (31)	33 (28)	13 (11)	8 (7)	27 (23)
Hindi	170 (59)	45 (26)	32 (19)	56 (33)	16 (10)	21 (12)
Total	288	82 (28)	65 (23)	69 (24)	24 (8)	48 (17)

## Discussion

We conducted this study to demonstrate feasibility of crowd sourcing methods in terms of reach (place/person distribution), richness in the idea (explored using qualitative approach) on household waste segregation an “online slogan contest.” Further this study provided us more understanding of conducting such studies, increased our experience and helped us in identifying methodological and operational challenges. Through crowdsourcing, we recruited 969 participants for this study. Additionally we are also assuming that through contest advertising and dissemination, other individuals were also engaged indirectly. The shared message in the form of a slogan encompassed a wide range of information regarding aspects of household waste segregation.

Our study findings shows that the slogan entries were received mostly from younger age population (one out of 2 entries were from participants aged less than 25 years). Likewise, similar studies have revealed a higher proportion of younger individuals being represented (28, 29). The reason for this could be attributed to the greater accessibility of technology among younger population compared to other age groups. It can be further presumed that a younger age group is likely to be more open to receiving online information on these and related topics (30). This young population can be the “Messenger for change” to improve household waste segregation (30).

In spite of advertising about the study in social media and being sent to relevant groups/institutions/universities across the country, the contest entries were mainly received from three states of Central and Western India. This may be due to the proximity of the organizing institutes that are located in Central part. Organizing institutes may be more known in this area compared to other parts of the country. Additional factors contributing to the increased number of entries from this region could be due to the presence of certain cities that have achieved higher rankings in the annual “Swachh Survekshan,” a government-led assessment of waste management in Urban India (31). The consistent promotion of waste segregation through awareness campaigns in these cities could be a contributing factor to attract a higher number of entries for the contest. The limited number of entries from non-Hindi speaking states might be attributed to the language restriction (only English and Hindi). The contest information for this contest was also shared in Hindi and English (and not in other regional languages) that may have further limited the participation. A research study on crowdsource-based contest in sexual health also highlighted the presence of a language barrier in the participants’ responses (32). From this study it is evident that in order to increase public participation in such crowdsourcing activities it is important to provide information/ publicize issues in local language (33).

The study received around 7 of every 10 entries from urban settings. This could be due to the digital divide. In urban settings, several factors play a role in enhancing participation, such as improved internet accessibility, greater knowledge and awareness, increased social engagement, and stronger connections through social media. In addition, the contest information was predominantly distributed among Municipalities, Swachh Survekshan cities, and prominent educational institutions, while Panchayats and villages received relatively limited attention. As a result, the contest details might not have reached rural areas as effectively, possibly contributing to the lower participation rates.

In our study, slogans from the contest highlighted different aspects of waste segregation, such as community awareness, need for waste segregation, use of different bins for different waste categories, citizen responsibilities, impact of waste segregation on the environment, and sustainability, among others. These aspects are crucial for achieving sustainable waste management. Adapting these domains into the waste management activities of urban local governments may help to enhance community awareness. According to research, crowdsourcing can be successfully utilized in a variety of other public issues as well (8). Therefore, the messaging can be translated into various languages in order to raise awareness, particularly in regions where waste management services are inaccessible. Messages from the contest can be used to educate communities and modify their perspective on waste segregation. The concept of crowdsourcing can also be applied to offline settings where internet access is limited, but it is rarely applied as it may require more logistical planning and resources to organize and manage compared to online crowdsourcing platforms (34, 35). Slogans received from the general public were used in designing the cover page of the intervention communication tool (flipbooks) of IMISS Project. The slogans were reviewed to select those that precisely represent the flipbook themes and were deemed appropriate for the flipbook content. The slogans chosen based on the themes of flipbook are detailed in Supplementary material 3.

The slogans provided valuable insights into how the general public perceives waste segregation, waste management, and environmental concerns related to waste. Many slogans conveyed a sense of civic and social responsibility toward waste segregation, serving as a motivational factor. Flipbook 2 of IMISS intervention highlighted the importance and benefits of household waste segregation. It is also indicated from the slogans that public have a limited understanding of other types of waste beyond dry and wet waste. Thus flipbook 3 was designed to create awareness about all types of wastes such as sanitary, hazardous, electronic, textile, food waste in addition to wet and dry waste. It was also observed from the slogans that there is a lack of public awareness about the health impacts of waste. Consequently, this aspect was utilized to raise awareness on the same. A table calendar was made as a nudge to remind the RCT intervention recipients about waste segregation and as a ready reference for categories of wastes, wherein some of the slogan were used in the design.

The study demonstrated that crowdsourcing is a feasible strategy to gather varied opinions and suggestions from a wide variety of people to come up with unique solutions to problems like solid waste management. Such online approaches may enhance public engagement in policymaking and good governance, promoting waste segregation awareness in the community, especially among younger people (36, 37). It is crucial to engage the community in meaningful connections and impactful research initiatives in order to promote waste segregation awareness (36).

Community engagement approaches traditionally have a narrow focus and are led by scientists whose perspectives may not align with those of the community (38). Additionally, standard community involvement initiatives may not reach potential stakeholders, who are generally disconnected from research (39). Crowdsourcing as a strategy at the local, state, and national levels can increase community participation in shaping policy decisions. Wazny (40) argues that crowdsourcing enables cost-effective community engagement, especially in low-resource environments, and that its transparency allows the public to address issues anonymously, resulting in a wide range of perspectives and enhancing intervention effectiveness.

The study’s strength was that it assessed the feasibility of the crowdsourcing method as an alternative approach to gather slogans to promote public awareness on improving household waste segregation. This study was a pioneer attempt to use the crowd sourcing strategy to gather slogans in the area of solid waste management, aiming to harness the collective creativity of the public for promoting sustainable waste practices. Through this study, we successfully disseminated the information to a wider group of people and obtained input from individuals spanning various age groups and regions within the country, which can be regarded as a notable advantage of the study.

The study invited slogans only in English and Hindi languages that might have resulted in fewer numbers of entries from non-Hindi speaking states. Furthermore, we could not reach out to individuals lacking access to digital platforms, which was noted as one of the limitations of the study.

## Conclusion

In this study, we have demonstrated the feasibility of crowd sourcing as a quick method to collect slogans/ideas/opinions about household waste segregation from a diverse population across the country. Authorities can use this approach to improve waste management approaches, since it involves a wider audience within a short amount of time. Crowdsourcing is gaining prominence as a prevalent method for collaborative and inventive resolution of problems. While it is still in its early stages, there is a clear need for more comprehensive research on the formulation of crowdsourcing tasks. Crowdsourcing can enhance community engagement with minimal cost, making it ideal for low-resource environments. Public input gives varied perspectives on issues and boosts intervention efficacy.

## Data availability statement

The original contributions presented in the study are included in the article/Supplementary material, further inquiries can be directed to the corresponding author.

## Author contributions

MK initiated the concept and formulated the initial design with VD, KK, and KS. KK, AS, AP, and VD coordinated the crowdsourcing activity. KK, KS, SS, KR, MK, and VD analyzed the data. KK and KS drafted the first version of the manuscript. KS, SS, KR, AP, CS, and SA provided the critical comments on the concept, design, and draft manuscript. All authors have commented on the earlier versions of the manuscript and read and approved the final version of the manuscript.

## Footnotes

^1^ imisswaste.rdgmc.edu.in

^2^ nireh.icmr.org.in
