# Supplementary material for: Feasibility assessment of crowdsourcing slogans for promoting household waste segregation in India: a cross-sectional study
Source: Front Public Health. 2023 Oct 11;11:1118331. doi: 10.3389/fpubh.2023.1118331 (PMC10600395; doi:10.3389/fpubh.2023.1118331)

<u>Supplementary Material 1</u> – <u>Contest Poster (English & Hindi)</u>

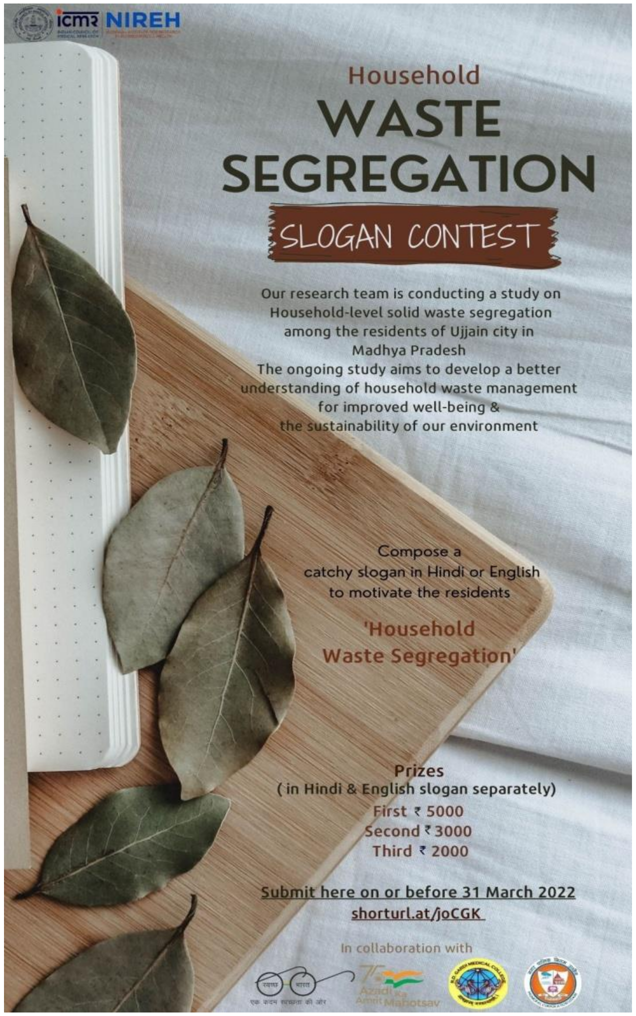

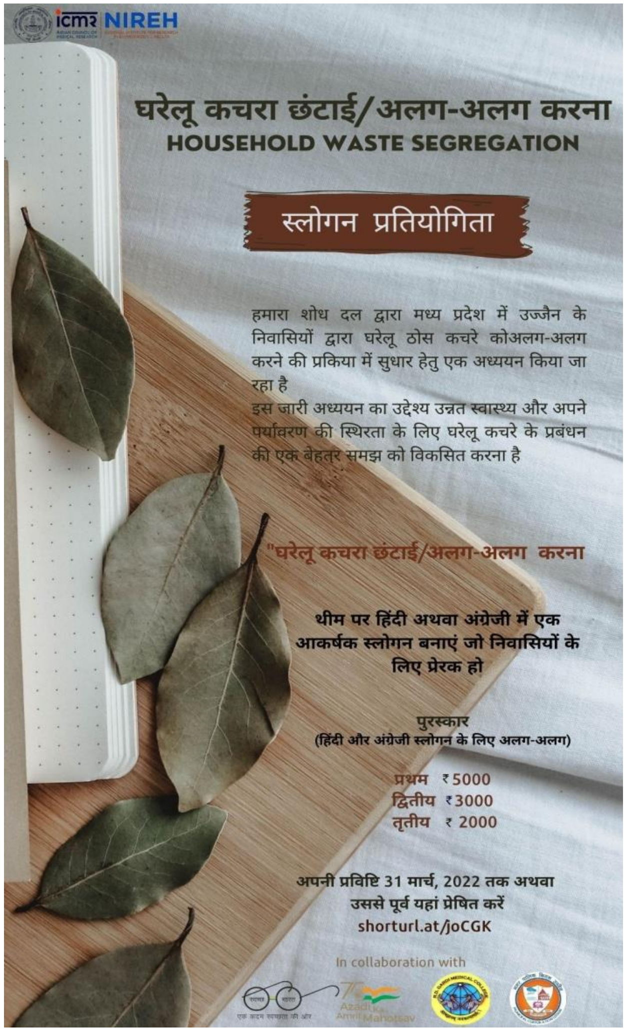

Supplementary Material-2

Lists of slogans in relation to household waste segregation in English and Hindi language

Thematic Areas	Community Awareness, Responsibility and Support	Significant of household waste segregation	Use of separate dustbin	Health and Wellbeing	Environment and sustainability
	**English language**<br><br>1. Segregate Wet Dry Waste Every day, Beautify Earth in Simple Way<br>2. Segregate your household waste properly,<br>Dry or wet, rubbish or garbage, dispose accordingly.<br>3. Start Segregate the Waste Perfectly! Get Integrate with Life Completely!!<br>4. "For Smart, Clean & Healthy Nation, Practice Household Waste Segregation."<br>5. Proper "Waste Segregation", leads to "Earth's new generation"<br>6. Don't be in a haste, Takeout time to Segregate Household Waste<br>7. Make a Habit of "waste segregation" duty -To maintain "Earth's Beauty"<br>8. "Three categories to segregate, responsibly dispose your waste"<br>9. Slow down, think and segregate, before it's too late<br>10. Paper or paste, segregate your house hold waste!<br>11. Small effort in sorting of waste, will give our kids future taste<br>12. Separate our household waste unite to make our community clean<br>13. Respect those hands, that segregates the waste from home-lands.<br>14. India's motto for garbage<br>Segregate and Manage,<br>Innovate and Leverage!!<br>15. Do segregate the waste,<br>To create a better place.<br>16. "Know the BINS and Teach your KINS"	**English language**<br><br>1. Solid, Dry & Sanitary, Segregation of Waste is Mandatory.<br>2. Household waste segregation at source! Generates cleanliness and income resource!!<br>3. Segregate at source, cause it's easy, once a pile, it's going to be queasy<br>4. Sort, Reduce, Recycle and Reuse Extract resources out of Refuse<br>5. " Waste isn't waste until we waste it, so best is to properly segregate it<br>6. Segregate your waste, before it's too late. Today well lived will make every tomorrow great!<br>7. Don't dump in haste. First, segregate the waste.<br>8. Segregation is the first step towards recycling and recycling is the first step towards development.<br>9. Household waste segregation<br>Do it for the future generation.<br>10. Segregate your waste before it's too late, or landfill would fall before your door!<br>11. Make great haste, segregate the waste.<br>12. "Separate your waste and scrap got erased"<br>13. Today your waste tomorrow their requirements, segregate it for recycling!	**English language**<br><br>1. Segregate, separate and refuse the refuse<br>also remember, two trash cans to use!<br>2. Dry waste in blue and wet in green,<br>simple mantra to keep environment clean!<br>3. Dispose Right, Win the Fight<br>4. Wet in green, dry in blue, will eliminate the garbage queue!<br>5. Make wet and dry waste segregate and stop your future from degrade.<br>6. Paste The Household Waste into The Right Place with Grace!<br>7. Segregate now.... appreciate later<br>8. "To keep the city beautiful and clean, use 3 dustbins."<br>9. Mix not, put dry and wet in separate baskets.<br>10. Let's segregate wastes from wastes altogether!<br>Disposing of wastes in different bins matters!<br>11. Give life to plants and cool the earth by separating wet wastes in green bins<br>12. Think Clean and Divide the Dustbins<br>13. Put Waste in The Right Place	**English language**<br><br>1. Once mixed, it can’t be fixed. Segregate it all, no matter how small!<br>2. Separate the waste, get the best<br>And if managed then life will be in rest.<br>3. Segregate the waste, be the part of best.<br>4. Segregate the waste, life should be best or else u will peace in rest<br>5. say no to mixed waste for a clean and green environment<br>6. Segregate your waste for having better taste<br>7. Mixing food waste and paper waste will burn our planet.<br>8. The Job: Household Waste Segregation.<br>The Salary: Disease Prevention,<br>Environmental Protection and Prosperity of Nation!<br><br>**Hindi language**<br><br>1. कचरे की छंटाई, घर की भलाई	**English language**<br><br>1. Sustain your own nation - by practicing- household waste segregation.<br>2. Don't let the dry and wet waste merge, Segregate it on nature's urge!<br>3. To keep ecology integration, practice waste segregation<br>4. Segregation of Trash!!! Save Mother Earth from Crash!!!<br>5. Segregate-Segregate all Litter to Make Our Environment All Glitter<br>6. Shine the earth, segregation of waste is worth<br>Segregation at source, be the environmental force<br>7. Today we separate tomorrow we celebrate.<br>8. Segregate the waste and make the world a better place<br>9. Healthier earth in your hands, stop dumping and start a separating your trash. It's that simple.<br>10. Segregate waste, adorn the land<br>Promote cleanliness, blazen the plan.<br>11. No segregation, much pollution

17. Dump the waste according to bin,
To make the country green and clean.
18. Separate the house waste,
Make public participation best.
19. Be responsible segregate household waste at right place today for a better environment tomorrow
20. Be smart to segregate, not dumb to integrate
21. Garbage segregation answer to a neat and beautiful nation.
22. Donâ€™t Shy; Recycle the Dry!!
Never Regret; Compost the Wet!!
23. Litter only after waste segregation, it makes the world better.
24. " Segregation would be more, India would be cleanest more "
25. To improve the planet future, sort your waste before dumping.
26. Segregate Waste to take rest
27. Divide and you will Rule... But this time, only for the good.
28. Think best.,
for the easy segregation of our household waste.
29. Let's aggregate to segregate.
30. Household waste segregation is better than cleanliness anticipation - to make it wow let's start now
31. Let's Collaborate & Segregate Wet and Dry Waste to Make Clean and Green Ujjain....
32. Waste may be dry or wet, but please segregate...
33. Segregate Waste for Ujjain- today and tomorrow!
34. Let us Segregate waste,
To Make Ujjain clean, green & best
35. Dear kith and kin, learn what to discard in which bin!
36. Don't discriminate among caste, but among waste
37. North south east west
Segregate your wet dry waste

14. Change the household waste to worth by segregating it and make it reusable again.
15. "Dry waste recycle, wet waste compost,
prohibit household trash mixing"
16. Segregate waste- hand over biodegradable to nature, man-made to machines
17. Segregate waste today to generate energy tomorrow
18. Segregate the waste before it segregates people.
19. Separation is the only way to transform my waste into wealth
20. Separate waste before you dispose to decorate your house with beautiful roses
21. Divide the waste, Earn, save our environment!
22. Segregation can be done, green composting should main.
23. Today we segregate, tomorrow we celebrate.
24. Charity begins at home, so does waste segregation, be the change you want the world to be
25. One man's leftover is another man's food! Segregate! Feed!
26. Waste Segregation: Every waste is different
27. Wealth cannot become waste but waste segregation converts waste into wealth.
28. If you think best household waste management is the best
29. *Not segregated yet!?Then refuse to waste.
*Don't Breathe, till Composting
*Either Separate or stop making wastes!
30. HOUSEHOLD WASTE IS NOT A WASTE BUT WEALTH TO WORLD
31. Please, don't mess up their Destinations!!

Hindi language

1. कचरे का है एक ही काट, गीले-सूखे में उसे दो तुम बांट।
2. घरेलूकचरे की करो छंटाई, तभी होगा पूर्ण सफाई
3. एकपहल स्वच्छ भारत के लिए गीला- हरा, सूखा- नीले के लिए।
4. होरही है सफाई की बात क्यों ना करे घर में दो बिन से शुरुआत |
5. यदि स्वछता का हैइरादा करो घर में दो बिन का वादा।
6. हरे - नीले में हीकरो निस्तारण, कचरा निवारण से सुरक्षित रहे पर्यावरण" II
7. स्वच्छताके लिये सबको करना होगा सजग, गीले - सूखे कचरे को रखना होगा अलग - अलग
8. हरघर में जगाएंगे उमंग, सूखा-गीला कचरा अलग-अलग रखकर, स्वच्छ बनाएंगे देश संग।
9. घर की स्वच्छता कारखिए ध्यान अपनाइए दो बिन, करिए देश का उत्थान |
10. कचरा प्रबंधन का होएक ही नारा गीले- सूखे कचरे का हो बँटवारा
11. जन-जनकी है पुकार, सूखे और गीले कचरे को, अलग करो अबकी बार।
12. Neele me Sookha,
Hare me Geela,
Naa banaye Kachre ka Teela!
13. स्वच्छता का वादाअटल, घर में दो बिन करे सफल।
14. गीला सुखा कचरा अलगकरो!
धरती को अपनी स्वच्छ रखो!
15. देश की प्रगति मेंहाथ बढ़ायेंगे , सूखा गीला कचरा अलग अलग रखवाएंगे

2. हम उत्पन करे घरेलुकचरा छटाई करे कतरा कतरा
3. करो घरेलु गीला सूखाहानिकारक कचरा की उचित छटाई तभी होगा स्वच्छ पर्यावरण की भलाई
4. घरेलू कचरा छटाईबीमारी महामारी भगाई
5. अपने घर का कचराछाँटो, मोहल्ले में खुशियाँ बाँटो।
6. गिला सूखा कूड़ा नहींमिलाएंगे ! आने वाली पीढ़ी का स्वास्थ्य बचाएंगे !
7. "कचरा प्रबंधनहो सबकी जिम्मेदारी, स्वच्छ बने फिर यह दुनिया सारी ।"
8. कूडे का निस्तारण होसाफ सुथरा घर आंगन हो
9. घर का कचरा रोगबराबर, कचरा गाड़ी का करो प्रयोग बराबर|
10. बेकार कचरा कूड़ा काउचित उपचार। स्वास्थ्य समृद्धि का अमूल्य उपहार।।
11. अगर चाहते होबीमारियों से दूरी, तो घर केकचरे को व्यवस्थित जगह रखना है जरूरी
12. देश का कचरा उतपन्नहोता है प्रतेक घर से साफ सफाई करो अपने हृदय से!
13. अपशिष्ट पृथक्करणकुंजी है स्वस्थ गृहस्थी और सुखी जीवन की
14. जनहित मे करदो जारीकूड़ाकरकट फैलाए बिमारी.
15. Nirdharit sthan pr kachra dale jeevan ko swasth banain
16. गीला - सूखा अलग करो ll
जीवन को सरल करो lll

Much segregation, no pollution.
12. Try to separate household waste,
Or it will get you in the hilarious state.
13. Don't to be late, do segregate!!
14. " Household Waste Segregation Practices for Clean, Healthy & Smart Nation "
15. Source segregation - Cleanliness to the nation
16. Segregate your waste and protect our earth.
17. SEPERATE WASTE - GENERATE NEW GREEN EARTH
18. East or West waste segregation is the best
19. To keep urself & environment happy, DO WASTE SEGREGATION!
20. Forests are Green,
Oceans are Blue;
Segregate the Wet-Dry Waste for Me and You.
21. "Keep in mind waste segregation, aim towards a sustainable nation"
22. Segregate Waste at Home Daily
Makes the Environment
Clean and Ready.
23. Clean Earth Green Earth dispose garbage in dustbin
24. Today If you do not segregate waste,
Tomorrow our planet may close the gate for our kids
25. Nature's force
Segregation at Source

Hindi language

1. साफ - स्वच्छ होगा आंगन अपना घर में दो बिन करेंगे पूरा ये सपना |
2. सबको मिलकर यह रायदो गीला कचरा सूखा कचरा अलग-अलग बांट दो
3. हिंदुस्तान हैहरियाला, बनेगा सबसे न्यारा, कचरा छटनी हो जन जन का नारा..
4. अपने घर का कचड़ा , अपनी जिमेदारी । कर सुखा-गिला कचड़ा अलग , है निभानी |
5. घर में पहुचना है ये ज्ञान, सुखे और गिले कचरे का हो अलग- अलगसमाधान।
6. अलग अलग डाले कचरासारा ताकी स्वच्छ रहे देश हमारा
7. हम सबकी हैजिम्मेदारी , घर घर से हो कचरा पूर्ण निस्तारित ।
8. घर में रखें दोकूड़ेदान क्योंकि सुखा गीला नही समान।
9. सूखा-गीला कचरा कीकरें छटाई | प्रबंधन ज्ञान और घर की सफाई ||
10. गीला ,सूखा कचरा करोअलग... स्वछता के प्रति रहो सजग....
11. बुद्धिमान सूखे औरगीले कचरे को करें अलग अलग, क्या आप हैं बुद्धिमान ?
12. हम उत्पन करे घरेलुकचरा छटाई करे कतरा - कतरा
13. घरेलू कचरे कीछंटाई, है हम सब की सामूहिक जिम्मेदारी।
14. कूड़े की छटनी करकेपर्यावरण सवचछ बनाएँ, आओ हम सब मिलकर अपनी अपनी जिम्मेदारी निभाऍ
15. "बच्चेंमहिलाये कदम बढ़ाये, घरेलु कचरा छटाई अभियान को सफल बनाये"
16. कचरा अलग करकेदीजिये, जनभागीदारी कीजिये
17. कूड़ा कूड़ेदान मेंडालो सूखा गीला देखकर डालो
18. Aaj kachre ka batwara banaye hamara bhavish nayara
19. बनाना हो स्वस्थसमुन्नत देश, सही कचरा प्रबंधन रखो उद्देश्य..
20. Geela, sukha kachra alagh rakhiye, ghar,parivar aur desh ko swasthya banaiye

32. Micro wastes contribute to larger wastes let us waste a minute to throw it carefully
33. Trash, trash, take a rest... And just segregate ....

Hindi language

1. कचरा अलग कराडाला, तो दुनिया झिंगालाला !!
2. जब होगी कचरे कीछटाई, तभी यह कहलाएगी पूर्ण सफाई।
3. घर में ही करें अपनेकूड़े को अलग सफाई का ये मंत्र है कितना सुलभ |
4. Gila sukha hain Dushman, karo alag yehi hain mission.
5. Jab kachra hoga prithak, tabhi safai hogi sarthak.
6. हरकचरा न एक समान। इस देश का रखना हमको ध्यान।।
7. एकदिन कचड़ा भी सोना उगलेगा , जब सुचारू निष्पादन होगा ।।
8. एकप्यारा सा बंधन, घरेलू कचरा प्रबंधन।
9. हम सब कोजागरूक रहना है, कचरा छांटकर फेंकना है।
10. "अलग-अलग कचरेका रखरखाव I स्वच्छ घर और नया उत्पाद I I"
11. कचरे का कर उचितनिपटान, विश्व में हो अपनी पहचान।
12. कचरे को रखो छांटहमेशा घर रहेगा दमकता हमेशा
13. घर से कचरा अलगकरेंगे, धरती कि हम रक्षा करेंगे।
14. खुशियोंका खोले पिटारा , घरेलु कचरे का हो उचित निपटारा।
15. अपनेघर के कचरे का सही करे निपटान अपना व अपने परिवार की बनाए शान
16. घरेलूकचरा, कर ले छटाई मोटा कचरा बेच कर ले कमाई
16. समस्त जनों सुनोमहत्त्व कचरे दान का 4 bin आवश्यक है स्वच्छ अभियान का.
17. सूखे-गीले कचरे मेंफर्क करे। अपने परिसर को स्वर्ग करे ।।
18. अलग करो सूखा और गीला कचरा उद्योग बढाओ, खाद बनाओ, करदो भारत हरा भरा
19. Swachhata Karmi ka ka Sahyog Karen Gila Sukha kachra Alag Alag Karen
20. "चार भागों मेंकरें छटाई| गीला, सूखा, कांच, पॉलिथीन अलग-अलग भगाई"||
21. "Geela--Sukha--kachra alag kareeye , Dhartee maa ke madat kareye"
22. सिर्फएक आदत कचरामुक्त भारतकी स्रोतपे कूड़ा अलग अलग करनेकी ।
23. यदि पर्यावरण को हैबचाना, घरेलू कचरा छंटाई ज़रूर अपनाना।
24. गीले और सूखे कचरेको अलग करो, अपने बच्चों का जीवन सफल करो।
25. कचरे की करो छटाईगिला कचरा सूखा कचरा अलग अलग करो भोजाई
26. सुंदरस्वच्छ भारत हमारा सपना, गीला सूखा कूड़ा अलग-अलग रखना।
27. गीलेसूखे में बांटेंगे, कचरे को अब छांटेंगे,,
28. घर को रखें साफ, गिले और सूखे कचरे को अलग अलग से फेंके आप ।
29. सूखा और गिला कचराअलग - अलग रखना एक ज़िम्मेदारी समझे, काम नहीं
30. गीलेकचरे से खाद, सूखे का उपयोग I हरी-भरी धरती करके, फैलाओ उद्योग II

26. Give life to plants and cool the earth by separating wet wastes in green bins
27. Don't throw the out the garbage otherwise earth give you savage

Hindi language

1. छंटाई करके ही कूड़ा डालें !स्वच्छ रहेंगी गलियां और नदियाँ -नाले!!
2. Kachare Ko Sahi Dibbe Mein Daalna Huzoor, Jannat Tho Khuljayega Idhar Hi Zaroor
3. कचरे का हो उचितप्रबंध, सुधरे मानव व पर्यावरणीय संबंध।
4. घरेलु कचरा फेंकतेसमय रहे ध्यान गीला एवं सूखा कचरा न हो एक साथ।
5. घर का कचरा एक मेंनही हे मिलाना , पर्यावरण हमें हे बचाना
6. छाटो कचरा, चुनोसफाई पर्यावरण की करो भलाई
7. "इस धरती कोही स्वर्ग बनाओ.... घरेलू कचरा छटाई प्रबंधन अपनाओ"!
8. Kachra prabandhan apnayen , Prakriti Sanrakshan paye|
9. कचरे का सहीनिस्तारण करना है पर्यावरण को स्वच्छ करना है
10. गीला-सूखा अलग करो ।धरती पर बोझ कम करो।।
11. सूखा और गीला कचराअलग अलग रखें। पर्यावरण मित्र और हितैषी बनें।।

21. घरेलू कचरे का करे वियोग, देश को मिलेगा सहयोग।
22. करें अलग गीला सूखाकचरा बनायें राष्ट्र स्वच्छ सुंदर हरा भरा
23. कचरे को गीला सूखाबाटेंगे, अपने शहर/देश को स्वच्छ बनाएंगे
24. सूखे गीले कचरे काकरे निदान , भारत देश का हो कल्याण।
25. Agar banana hai bharat ko Mahan, toh kachara alag karne mei hi h shaan
26. गिला सूखा कचरा अलगअलग रखना भाई तब होगा गांव स्वच्छ
27. Kuda alag alag kar k he dena hai bharat ko swach bnana hai
28. कचरा प्रबंधन घर बनेमधुबन
29. "अगर स्वच्छरहना है हमको, कचरा प्रबंधन करना है हमको"
30. गीला कचरा अलग , सूखा कचरा अलग ... हर उज्जैनवासी में जगे ये अलख ...
31. अलग अलग निकाले कचरासूखा और गीला तभी उज्जैन बनेगा हरा- भरा और चमकीला
32. कचरा छांटो इंसाननहीं
33. कचरे को हटानाहै,भारत को स्वच्छ बनाना है
34. "बच्चो काभविष्य उज्ज्वल बनाना है, कचरे को सही जगह पहुंचाना है।"
35. कचड़ा फेलाने मैंभागीदारी है , अब निस्पादन की जिम्मेदारी है।।
36. जन जन ने ठाना हैकचरे को डस्टबिन में डालना है
37. Tera kachra Tu zimmedar
Geela Sookha alag kar yar
Prakriti ko bhi vyakt kar abhaar !
38. पर्यावरण कोबचायेंगे कचरा प्रबंधन हम अपनाएंगे
39. रोज़ करो कचरे की घरसे सफाई। इसी में है देश की भलाई ||
40. स्वच्छ राष्ट्रबनाना है,हमलोग को कचरा डस्टबिन में ही डालना है।
41. कूड़े कचरे का होखात्मा, यही कहे भारत की आत्मा।
42. पर्यावरण कोबचाएंगे कचरा प्रबंधन हम अपनाएंगे,कचरा प्रबंधन ना अपनाएंगे तो मुसीबतों को बढ़ाएंगे

17. हर कचरे पर लिखा हैउसका पता, आइए हम करें मदद ना हो वे लापता I
18. गीले सूखे कचरे कीछटाई है जरूरी ,सफाई है जरूरी
19. खाद बना गीले कचरेसे, सूखे का फिर उपयोग I पर्यावरण स्वच्छ करके, दूर भगाओ रोग II
20. घरों में गीला - सूखा अलग करो, गीले को खाद्य, सूखे को रीसाइकिल करो।।
21. कचरे को जड सेउकारना है देश को साफ बनाना है
22. अलग रखे हम कचरेको, रसोई के कचरे को खाद बनाये कागज को उपयोगी पदार्थ बनाये ।।
23. कचरा तो कचरा हैइसको यहाँ ना डालो वहाँ ना डालो, डालो तो लेकिन कचरा पात्र में डालो.
24. अगर निरोगी रहना हैंतो रखें घर आँगन स्वच्छ और कचरे का करें सही निदान ।
25. अगर धरती को हैंबचाना, कचरा प्रबंधन को हैं बढ़ाना।
26. "पर्यावरण सेअनुपयोगी कचरा हटाना है, जीवन में उपयोगी कचरा लाना है| "
27. आओ पर्यावरण स्वच्छरखें हम कचरा प्रबंधन करें
28. कचरा नहीं कंचन है, सूखा कर अलग गीला खुदबू अलग
29. गाड़ी वाला आया घर से कचरा निकाल, इकट्ठा नहीं, अलग अलग करके डाल।।
30. सरकार ने दो कूड़ेदानलगाए नीले हरे को देखकर अपनाएं
31. Jab gaadi wala Ghar se kachra nikal
32. **Agar banana hai bharat ko Mahan, toh kachara alag karne mei hi h shaan**

31. Ghar ka kachra hra dabba gila. Swasth ho jeevan sukha kachra neela.
32. घरेलू कचरे का यहीउपचार, सुखे -गिले का अलग संसार||
33. घरेलु कचरे का यहीनिदान,सुखे-गीले का भिन्न स्थान ||
34. गीले सूखे कचरे काकरो अलग समाधान, स्वच्छ और सुंदर बनेगा हिंदुस्तान।
35. स्वच्छताके हैं दो ही निदान, नीला और हरा कूड़ेदान
36. आओसब मिलकर शुरुवात करें गीले कचरे से खाद बनाएं सूखा कचरा अलग रखें
37. सूखा गीला और घरलू कचरा I छटाई करके सफाई से रहना I
38. घर को रखो स्वस्थगिला सुखा कचरा करो अलग
39. जब दो कचरा, कचरापेटी में। तब बांट दो कचरा, कचरा पेटी में।।
40. सूखा कचरा गीला कचराअलग करे, आगे बढ़ें।
41. गीला - सूखा कचरा कोपहचान, स्वच्छ भारत की यही है शान ।
42. इससमस्या को दूर करेंगे, कचरे को अलग-अलग डस्टबिन में रखेंगे।
43. हरदिन दो बिन
44. पर्यावरणको बचाएंगे कचरा प्रबंधन हम अपनाएंगे
45. सूखा - गीला कचरा, पृथक - पृथक रखना है। सूझ - बूझ से अपनी, पर्यावरण बचाना है।।
46. हमें गीले कचरे सूखे कचरे को ध्यान में रखते हुएवयवस्थीत जगह पर इकट्ठा करना चाहिए
47. घरमें रखें तीन डस्टबिन, गिला अलग, सुखा अलग और हानिकारक कचरा को रखे अलग।
48. गीले कचरे कोअलग और सूखे कचरे को अलग करना चाहिए

12. अपशिष्ट पृथ्थकरण सेहमारे देश के प्रगती को सराहना मिलती है
13. गीला - सूखा अलग करो ll जीवन को सरल करो lll
14. ।ही कचरा प्रबंधनअपनाएं, पर्यावरण बचाएं
15. गीला - सूखा अलग अलगहो कचरा । पर्यावरण - मानव को ना हो क्षति, कम हो ख़तरा ।
16. घर का कचरा बाहर नडालो, सफाई रख धरती की जान बचालो!
17. Kachre ko hatana hai, apne baccho ko swachacha Bharat dikhana hai.
18. हम सब पर्यावरणप्रदूषण से बचेंगे,जब ठोसअपशिष्ट का प्रबंधन एकत्रीकरण,पृथक्करण,पुनर्चक्रणके महत्व को समझेंगे
19. कचरेका करो निपटान, धरती का करो उत्थान
20. साथीरेहाथसेहाथमिलाना गंदगी को दूर भारत से है भगाना
21. अगर धरती को हैं बचाना, कचरा प्रबंधन को हैं बढ़ाना।

43. सफाई का है बड़ाअभियान, सभी दे इसमें अपना योगदान.

44. स्वच्छता की मशाल कोचहूँ ओर जलाना हैं। सूखा- गीला कचरा निस्तारण जीवन मे अपनाना हैं।।

45. "कचरा प्रबंधन हो सबकी जिम्मेदारी, स्वच्छ बने फिर यह दुनिया सारी । "

49. Gila sukha alag alag karle Bhai alag alag à¥¤

50. कचरे की करो छटांईगिला कचरा सूखा कचरा अलग अलग करो भोजाई ।

51. अलग-अलग होंकूड़ादान, स्वच्छ भारत बने महान। अपना अपना फ़र्ज़ निभाओ, गली मुहल्ला स्वच्छबनाओ।।

52. कूड़ादान - महा दान

53. सूखा-गीला, कचरा-कचरा नीला-हरा, नीला-हरा

54. चलोसफाई की आदत डालें गंदगी को कूड़े दान में डाले

55. Sadne do iss kachare ko lekin dharti ko nahi

56. कचरे को अलग करेज़िन्दगियों को जोड़े गीले और सूखे कचरे को एक साथ न छोड़े।

Supplementary Material 3:

Series of flipbooks, cover slogans in English & Hindi and t theme

Flipbook series	Theme of book & cover slogans	English Cover Slogan	Hindi Cover Slogan
Flipbook 1 – Basics of waste segregation (with Environmental benefits)	Benefit to planet Earth through waste segregation	Shine the Earth, Segregation of Waste is Worth!	*Sukhe Geele Kachre Mein Fark Karein, Apni Dharti Ko Swarg Karein* (Segregation of waste will create heaven on Earth)
Flipbook 2 – Why segregate waste? The big picture: personal, civic, and environmental benefits of waste segregation	Civic benefits and civic duties of waste segregation	Sustain Your Own Nation,<br><br>By Practicing Household Waste Segregation !	*Chaar Kachre Ho Alag Alag,*<br><br>*Har Ujjainwasi mein jage yeh alakh!* (An emotion of waste segregation in four categories should arise in hearts of every citizen of Ujjain)
Flipbook 3 – Types of waste & their disposal: in detail	Keeping waste segregated into different categories, to get benefits of resource recovery	Sort, Reduce, Recycle and Reuse,<br>Extract Resources out of Refuse !	*Ho Alag Alag Kachre Ka Rakhrakhaav, milein swachh ghar aur naye utpaad!* (Keep waste segregated, to get clean home and new products)

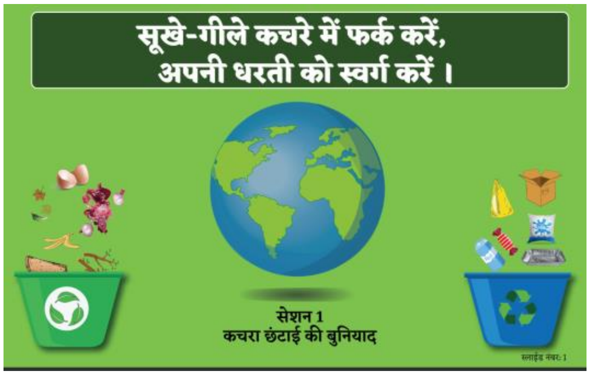

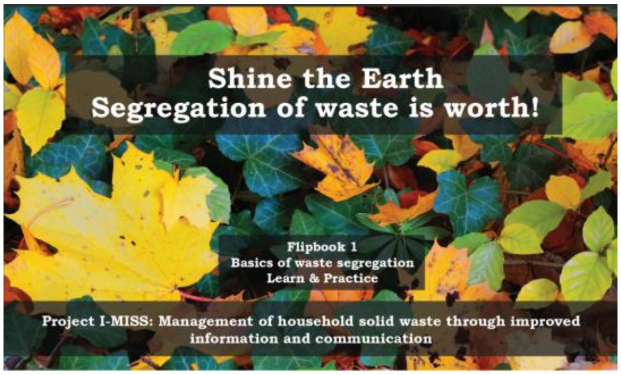

Supplement: Supplementary file 1 [file Table_1.pdf]
